# Early Neural Development of Social Interaction Perception: Evidence from Voxel-Wise Encoding in Young Children and Adults

**DOI:** 10.1523/JNEUROSCI.2284-23.2024

**Published:** 2024-10-28

**Authors:** Elizabeth Jiwon Im, Angira Shirahatti, Leyla Isik

**Affiliations:** Department of Cognitive Science, Johns Hopkins University, Baltimore, Maryland 21218

**Keywords:** development, fMRI encoding, social interaction

## Abstract

From a young age, children have advanced social perceptual and reasoning abilities. However, the neural development of these abilities is still poorly understood. To address this gap, we used fMRI data collected while 122 3–12-year-old children (64 females) and 33 adults (20 females) watched an engaging and socially rich movie to investigate how the cortical basis of social processing changes throughout development. We labeled the movie with visual and social features, including motion energy, presence of a face and a social interaction, theory of mind (ToM) events, valence, and arousal. Using a voxel-wise encoding model trained on these features, we found that models based on visual (motion energy) and social (faces, social interaction, ToM, valence, and arousal) features can both predict brain activity in children as young as 3 years old across the cortex, with particularly high predictivity in motion-selective middle temporal region and the superior temporal sulcus (STS). Furthermore, models based on individual social features showed that while there may be some development throughout childhood, social interaction information in the STS is present in children as young as 3 years old and appears adult-like by age 7. The current study, for the first time, links neural activity in children to predefined social features in a narrative movie and suggests social interaction perception is supported by early developing neural responses in the STS.

## Significance Statement

This study investigates the neural basis for social scene perception ability in children using fMRI data collected while participants watch a short, animated movie. Unlike most prior studies with movies, we labeled a range of visual and social features in the movie and used machine learning analyses to link each feature to fMRI responses in adults and children ages 3–12. Notably, our results demonstrate strong evidence that children as young as 3 years old show significant responses to most visual and social features in the movie, including social interaction responses in the superior temporal sulcus, a region in the brain that is well known to be important in social interaction processing in adults.

## Introduction

Behavioral studies have demonstrated that very young children have advanced social perceptual abilities, including recognizing social interactions and extracting complex concepts about other agents and their relationships from these interactions (Hamlin et al., 2007; Powell and Spelke, 2018; Goupil et al., 2022; Thomas et al., 2022). A growing body of work suggests that social interaction perception is a core human ability that is separate from other forms of social processing like face recognition or theory of mind (ToM; McMahon and Isik, 2023). In adults, neuroimaging studies have shown that regions in the superior temporal sulcus (STS) process others’ social interactions, in a manner that is distinct from other social and visual information such as motion, faces, or ToM (Isik et al., 2017; Walbrin et al., 2018; Lee Masson and Isik, 2021). Despite young children's advanced social perceptual abilities, it has been difficult to study the development of social interaction selectivity in the brain due to challenges in scanning young children. One recent study has identified social interaction selectivity in the STS in children as young as 6 but with reduced selectivity compared with adults (Walbrin et al., 2020). Another recent study with fNIRS (functional near-infrared spectroscopy) has shown that in infants, the medial prefrontal cortex (mPFC) and STS both show stronger responses to interacting than noninteracting dyads, but responses in the STS were similar for upright and inverted dyads (Farris et al., 2022), leaving open the question of whether the STS is selective for social interactions in children under 6.

In recent years, naturalistic or narrative stimuli have been gaining popularity in neuroimaging studies due to their more dynamic and engaging nature compared with traditional static stimuli (Redcay and Moraczewski, 2020). These methods are particularly promising for child neuroimaging studies (Vanderwal et al., 2019) and have yielded insights into the neural development of cognitive functions ranging from mathematics (Cantlon and Li, 2013) to social cognition. For example, Richardson et al. (2018) scanned an impressive number of 122 3–8-year-old children while they viewed a Pixar short film and used inter-region correlation (IRC also known as functional connectivity) to show that brain regions in the ToM and pain networks are functionally distinct by age 3 and that functional specialization increases with age. Like this example, many studies that use movie stimuli use IRC or intersubject correlation (ISC) to allow neural patterns to emerge from rich datasets (Redcay and Moraczewski, 2020). However, one limitation of these methods is that it is difficult to tell what movie content is driving the correlated neural activity.

To study young children's brain responses to social interactions during movie viewing, we used an approach that has been effective in adult neuroimaging studies, combining dense movie labeling of social and visual features with voxel-wise encoding (Lee Masson and Isik, 2021). We applied these methods to the fMRI data from Richardson et al. (2018) and found that encoding models based on visual and social-affective features could explain neural responses in children as young as 3 years old. Notably, we also found evidence specifically for social interaction responses in the STS in children as young as 3, with only minimal differences between young children and adults.

## Materials and Methods

### fMRI data and preprocessing

We analyzed a publicly available fMRI dataset from Richardson et al. (2018). There was a total of 155 participants, including 122 children [3–4 years (*n* = 31), 5 years (*n* = 34), 7 years (*n* = 23), 8–12 years (*n* = 34); 64 females] and 33 adults with ages ranging from 18 to 39 years [M(SD) = 24.8(5.3); 20 females]. The participants watched a 5.6 min animated Pixar movie, Partly Cloudy (Sohn, 2009), presented without sound. Full details of the data collection and preprocessing can be found in the original paper (Richardson et al., 2018). Briefly, participants’ MRI images were registered to the Montreal Neurological Institute (MNI) template and visually inspected to ensure proper alignment of key anatomical landmarks. Data were next smoothed with a 5 mm kernel Gaussian filter. Artifact time points were detected if there was >2 mm composite motion between time points or a fluctuation in global signal that exceeded 3 SD from the mean global signal. The original authors excluded participants with >1/3 artifact time points, and they are not included in our analyses. For remaining subjects included here, there was a difference between the number of artifacts in adults and children, but no differences among the different child age groups, suggesting differences across children cannot be attributed to differing levels of motion. PCA (principal component analysis)-based CompCor noise regressors were calculated in individual subject white matter masks from the scrubbed data (after artifact time point removal and interpolation). Both artifact time points and CompCor noise regressors are included with the publicly available data and were regressed out before encoding analysis.

In addition to whole-brain analyses, we also analyzed data in two regions of interest (ROIs): middle temporal region (MT), which is known to be involved in visual motion processing, and the STS, a region known to be involved in social processing. For MT, we used an anatomical mask provided by Wang et al. (2014). For STS, we used an anatomical mask from Deen et al. (2015) that includes parcels for both the temporoparietal junction (TPJ) and STS, regions known to be involved in ToM and social perception, respectively. Both anatomical masks were developed based on adult brains, but due to the relatively low spatial resolution of fMRI, previous work has supported the use of a common anatomical template for children this age, and anatomical registration was visually inspected for each subject by the original authors (Richardson et al., 2018).

### Movie annotations

To understand which features drove neural activity during movie viewing, we labeled different visual and social features of the movie. The movie was segmented into 2 s clips corresponding to TR length, resulting in 168 segments total. The opening (Segments 0–10) and closing (Segments 162–168) credits were trimmed from the labeling set. We automatically extracted visual motion features for each segment using an Adelson and Bergen motion energy model (Adelson and Bergen, 1985) implemented in the pymoten software package (Nunez-Elizalde et al., 2021). We used PCA to reduce the dimensionality of these features to 19 components, which explained ∼75% of variance in the data.

To label social and affective features in the movie, two authors (A.S. and L.I.) and one additional lab member watched the entire movie and then labeled the following features for each 2 s segment: the presence of faces, social interaction, and ToM events. Two raters also labeled valence and arousal for each 2 s segment. The annotators labeled a segment as having faces when the first frame of the clip contained a face; labeled a social interaction when two agents were acting in a directed manner toward each other or communicating with each other; labeled ToM events when one of the characters appeared to be thinking about the thoughts, emotions, or goals of another character (this “second-order” measure of ToM was designed to be more objective and tested in our prior study; Lee Masson and Isik 2021); labeled valence on a 1–5 Likert scale (very unpleasant, unpleasant, neutral, pleasant, very pleasant); and labeled arousal on a 1–5 Likert scale (very calming, calming, neutral, exciting, very exciting). These labels and scales were selected based on our prior work with movie encoding models in adult fMRI movie data (Lee Masson and Isik, 2021).

We also looked at how correlated the visual and social features were to each other using representational similarity analysis (RSA; Kriegeskorte et al., 2008). To construct the representational dissimilarity matrices (RDMs) for the visual features, we used Pearson's correlation distance (1, Pearson's *r*) between every pair of movie segment. For our social features, which were one-dimensional, we calculated the difference between mean ratings for every pair of movie segments. To compare each pair of features, we correlated the lower triangulars of each pair of feature RDMs.

### ISC

To identify voxels in each subject with consistent movie responses across the group, we used ISC to generate a whole-brain mask. ISC is a common method in fMRI to pinpoint voxels exhibiting reliable stimulus-evoked responses, excluding those that are unique or noisy (Nastase et al., 2019). We calculated the averaged Fisher’s *Z* transformed leave-one-subject-out correlation for each voxel and created the ISC mask by selecting voxels with a significantly above-chance (FDR correct *p* < 0.05) ISC value using the Brain Imaging Analysis Kit [BrainIAK (Kumar et al., 2021), version 0.1.1, “isc” and “permutation_isc” functions].

### Encoding model

**Figure 1.**
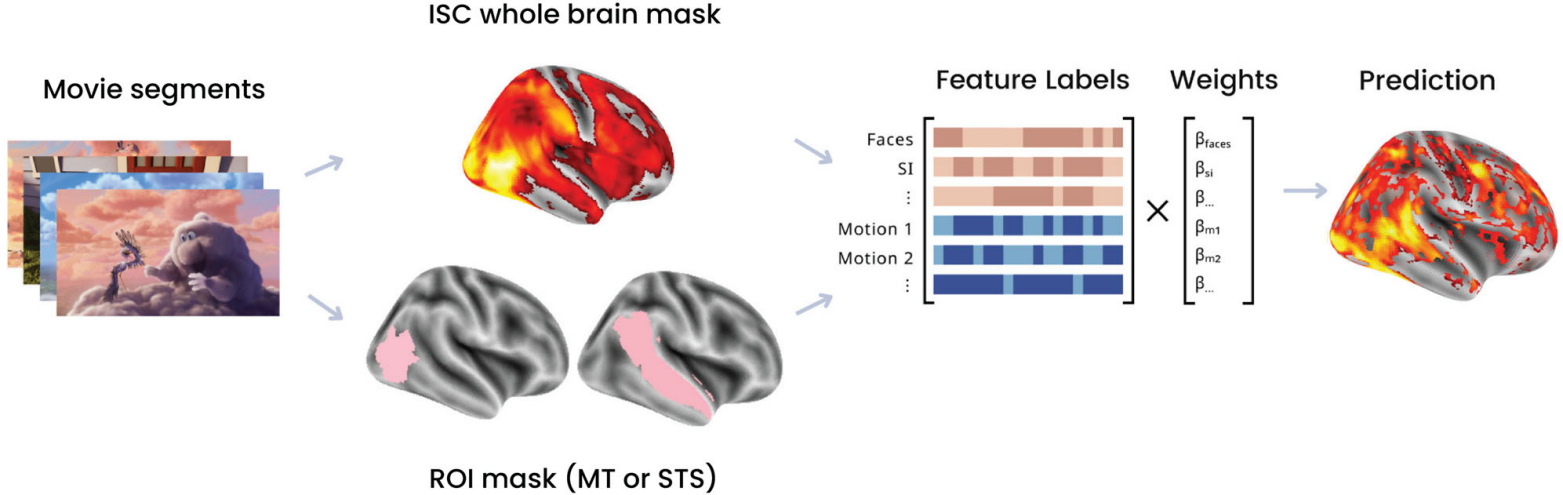
Schematic description of voxel-wise encoding model. We used an encoding model approach to predict voxel-wise responses to held-out movie segments in both whole-brain (thresholded with an ISC mask) and ROI (MT and STS) analyses. We labeled movie clips with visual (motion energy) and social features (the presence/absence of faces, the presence/absence of social interactions, the presence/absence of agent mentalizing, valence, and arousal). During the training, the model learned a set of beta weights to link the features to fMRI BOLD responses in each voxel over time. In testing, the learned beta weights were multiplied by movie features to predict the activity in the held-out fMRI data. The predictions were then compared with the true BOLD responses using a Pearson’s correlation score (*r*) that reflects the predictive accuracy of the model.

To relate brain activity to specific movie features, we trained a voxel-wise encoding model to learn a set of beta weights linking the movie features to fMRI BOLD responses (Fig. 1). Specifically, we learn a linear combination of feature weights to describe each voxel's activity. We used banded ridge regression (Nunez-Elizalde et al., 2019; Dupré la Tour et al., 2022), which is similar to classic ridge regression, but allows different feature spaces to have different regularization penalties. This is important for feature spaces with different dimensionality and with multiple collinearities. We learned one penalty term for the visual features and a second penalty term for the social features. Our prior work has shown that this is an effective way to account for the differing dimensionality across rich, high-dimensional visual features and simpler social-affective annotations (Lee Masson and Isik, 2021).

To account for temporal autocorrelations in the movie and fMRI responses, we divided the movie into 19 continuous segments (eight TRs each) and performed leave-one-out cross–validation on these continuous segments. We shifted the time course of our fMRI data by two TRs (4 s total) to account for hemodynamic lag and normalized the fMRI BOLD responses (*Y*) and features labels (*X*) by *Z*-scoring with the mean and standard deviation in the training set. The features were then split into two groups: one for the visual motion energy features (*X*_1_) and a second for the human-annotated social features (*X*_2_). The banded ridge regression is then defined as the following:

$$Y\;=\;X_{1}\beta_{1}+\;X_{2}\beta_{2\;\;\;}+\;\varepsilon ,$$

where $\beta_{1}$ and $\beta_{2\;\;\;}$are arrays containing the beta weights for each feature.

$${\hat{\beta }}_{\mathrm{banded}\;\mathrm{ridge}\;\;}=[\begin{array}{c} {\hat{\beta }}_{1}\\ \;{\hat{\beta }}_{2} \end{array}]={\left(\left[\begin{array}{cc} X_{1}^{T}X_{1} & X_{1}^{T}X_{2}\\ X_{2}^{T}X_{1} & X_{2}^{T}X_{2} \end{array}\right]+\left[\begin{array}{cc} \lambda_{1}^{2}I_{F1} & 0\\ 0 & \lambda_{2}^{2}I_{F2} \end{array}\right]\right)}^{-1}\left[\begin{array}{c} X_{1}^{T}X_{1}\\ X_{1}^{T}X_{1} \end{array}\right]\mathrm{Yw}.$$

Next, we predicted the brain response to held-out movie data ( $\hat{\Upsilon }$) by multiplying the features (*X*) of the movie with their relevant beta weights. We then compared these predictions with the true held-out neural responses recorded when participants watched the film using Pearson's correlation (*r*) which serves as our prediction score as follows:

$$\hat{\Upsilon }=X{\hat{\beta }}_{\mathrm{banded}\;\mathrm{ridge}}.$$

We fit our encoding model with all labeled features and then tested it with different subsets of features by setting the learned beta weights for other features to 0, to understand the different contribution of each individual feature or feature set, while controlling for the effects of covarying features. For ROI results, we perform encoding voxel-wise and then average the scores for all voxels in each ROI.

### Statistical analyses

To assess statistical significance, we used nonparametric permutation-based statistical tests. To compare encoding results in each age group to chance, we ran a sign permutation test with 5,000 resamples. Next, to compare each child age group to adults, we created a null distribution by randomly shuffling group labels (between child and adult groups) and calculating the difference between shuffled means 5,000 times. True group differences were compared with the null distribution to assess significance. For whole-brain results, voxel-wise responses were FDR corrected using the Benjamini–Hochberg procedure.

## Results

### Social features are represented throughout the movie and only minimally correlated with visual features

**Figure 2.**
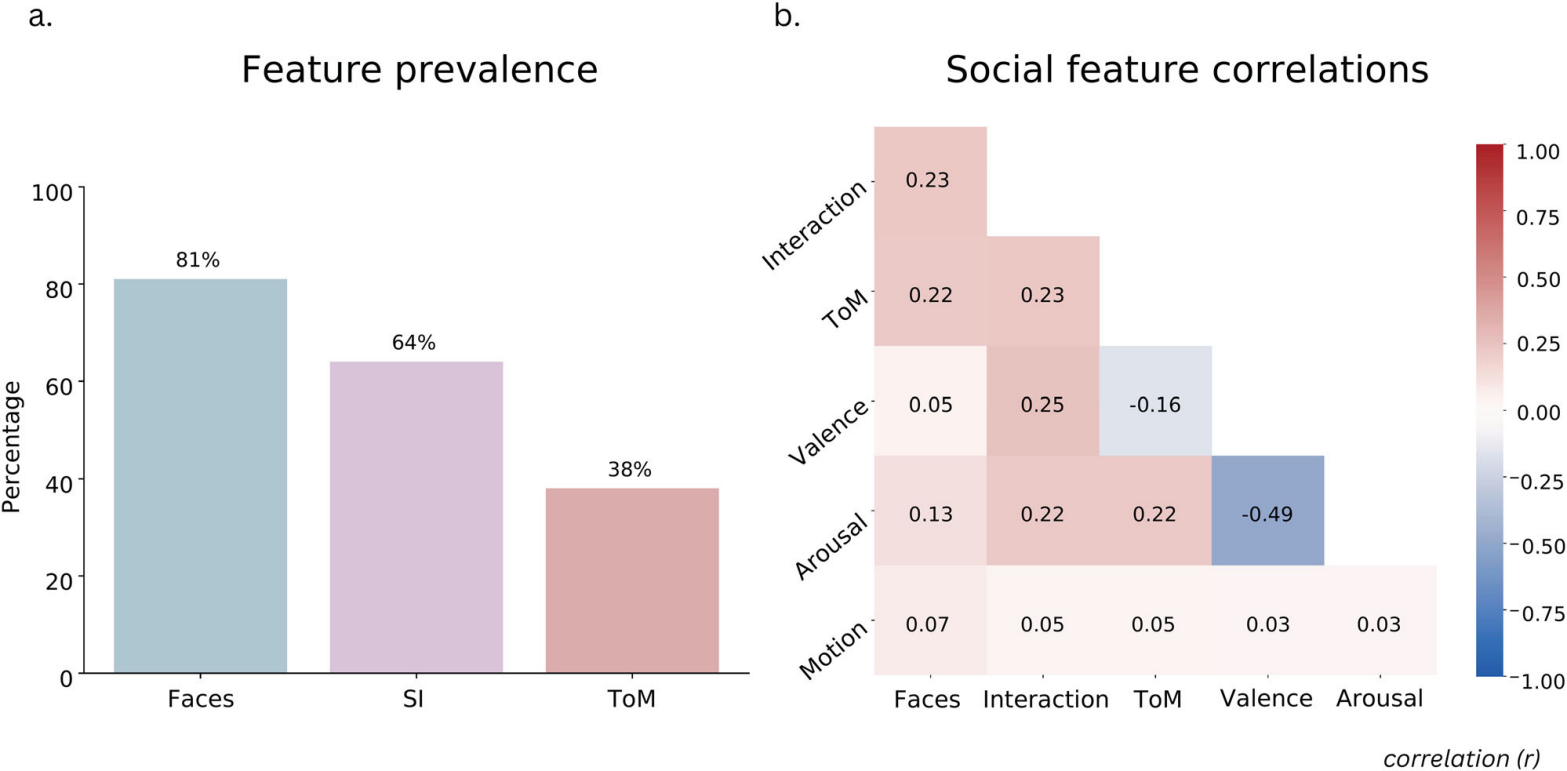
Feature prevalence and correlations. ***a***, Prevalence of key features throughout the movie. The *y*-axis shows the percentage of movie scenes that contained each of three key social features: faces, social interaction, ToM. ***b***, Feature correlations calculated using RSA between every pair of labeled features in the movie. Scores represent Pearson's *r* values between the lower triangular matrices of each pair of features RDMs.

To understand how social information was distributed across the movie, we looked at the prevalence of each of our labeled features throughout the movie (Fig. 2*a*). The average pairwise inter-rater agreement was 100% for faces, 71% for social interaction, 53% for ToM, 74% for valence, and 73% for arousal. We found that 81% of the movie scenes had faces, 64% of the movie scenes had social interactions, and 38% of the movie scenes had ToM events, suggesting that the social features were well represented in the movie. We also calculated the feature correlations with RSA to ensure that we could parse apart the contribution of different features on model performance (Fig. 2*b*). We found that the different features were only slightly correlated across the movie. In particular, the motion features were largely uncorrelated with social features.

### Both visual and social features explain whole-brain voxel responses in children as young as 3

**Figure 3.**
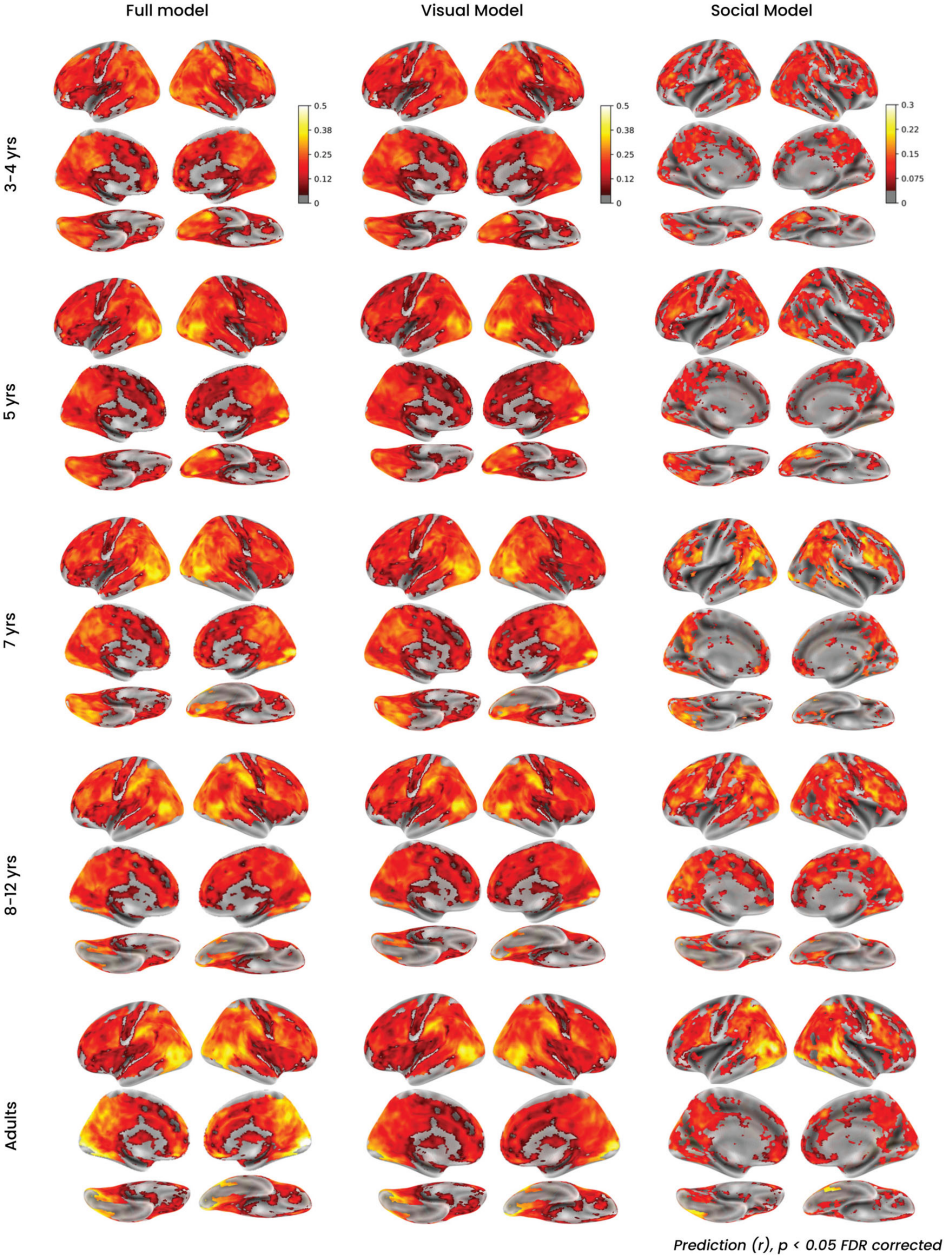
Whole-brain encoding model results. Average whole-brain prediction accuracy (Pearson's *r*) of three models using different subsets of beta weights for prediction—full model (left), motion energy only (middle), and social features only (right) for each age group: 3–4, 5, 7, and 8–12 years and adults. Results are mapped onto the MNI template and lateral (top), medial (middle), and ventral (bottom) views are shown on the inflated cortical surface.

We first conducted a whole-brain analysis using all features in our voxel-wise encoding model. We found that this model significantly predicted brain activity in the adult and all of the child groups, even in the youngest group of children (3–4 years old; Fig. 3, left). In exploratory analyses, we performed the encoding separately for the 3-year-old (*N* = 17) and 4-year-old (*N* = 14) age groups but observed lower group-level encoding scores and fewer significant voxels in each individual age group, likely because each analysis relies on about half the data as the combined group. Thus, we performed subsequent analyses on the combined 3–4-year-old group.

We also found above-chance encoding when only the visual motion energy (Fig. 3, middle) or social-affective features (Fig. 3, right) were used for prediction. Peak prediction for the full model was in the right early visual cortex (EVC) for all age groups ([10, −90, 0] for adults) and nearby for all age groups except 3–4 years, which showed a small cluster of highly predictive voxels in the frontal cortex but like all other age groups also had high predictivity in early visual and fusiform regions (Table 1). The visual model results looked very similar to those of the full model and had highest predictivity in ventral regions and posterior portions of the lateral occipital temporal cortex (LOTC), including MT with peak MNI coordinates in EVC for all age groups. These regions were also well predicted by the social model in all age groups, as well as posterior portions of the STS. Though there was some variability across age groups, the peak for all groups was in LOTC in close proximity to the adult peak, with the exception of 8–12-year-olds whose peak was in the mPFC.

**Table 1.** Peak MNI coordinates predicted by each model in the whole-brain ISC mask

	Full model	Visual model	Social model
3–4 years	[8, 64, −2]	[−22, 66, 2]	[42, −32, −18]
5 years	[−46, −76, 2]	[−48, −76, 2]	[−38, −46, −18]
7 years	[14, −92, 4]	[14, −92, 4]	[46, −34, 6]
8–12 years	[54, −64, 0]	[52, −64, 0]	[−16, 38, 42]
Adults	[10, −90, 0]	[10, −90, 0]	[−54, −70, 6]

Each row shows the MNI coordinate for the voxel best predicted by each model for each age group.

Encoding performance was high for all age groups, and a similar number of voxels achieved significantly above-chance performance in all child groups as adults for the full model and visual model (Table 2), suggesting that the encoding approach is robust in all age groups and that any other differences in predictivity across age cannot be attributed to lower data quality in the child groups. There were some differences across age groups for the social model where there are fewer significantly predictive voxels in children than adults (Table 2). However, a direct comparison between each age group and adults revealed no significant differences across age groups that survived whole-brain multiple–comparison correction.

**Table 2.** Significant voxels predicted by each model in the whole-brain ISC mask

	Full model (%)	Visual model (%)	Social model (%)
3–4 years	134,114 (98.2)	133,537 (97.7)	96,913 (70.9)
5 years	134,224 (98.2)	134,250 (98.3)	93,182 (68.2)
7 years	133,549 (97.8)	133,980 (98.1)	88,657 (64.9)
8–12 years	135,345 (99.1)	134,819 (98.7)	119,202 (87.3)
Adults	134,658 (98.6)	134,851 (98.7)	109,581 (80.2)

Each row shows the number of significant voxels predicted by different models for each age group. The numbers in parentheses indicate the percentage of the total adult ISC mask (136,617 voxels) that is significantly predicted.

### Visual and social features are represented in MT and STS in all groups

**Figure 4.**
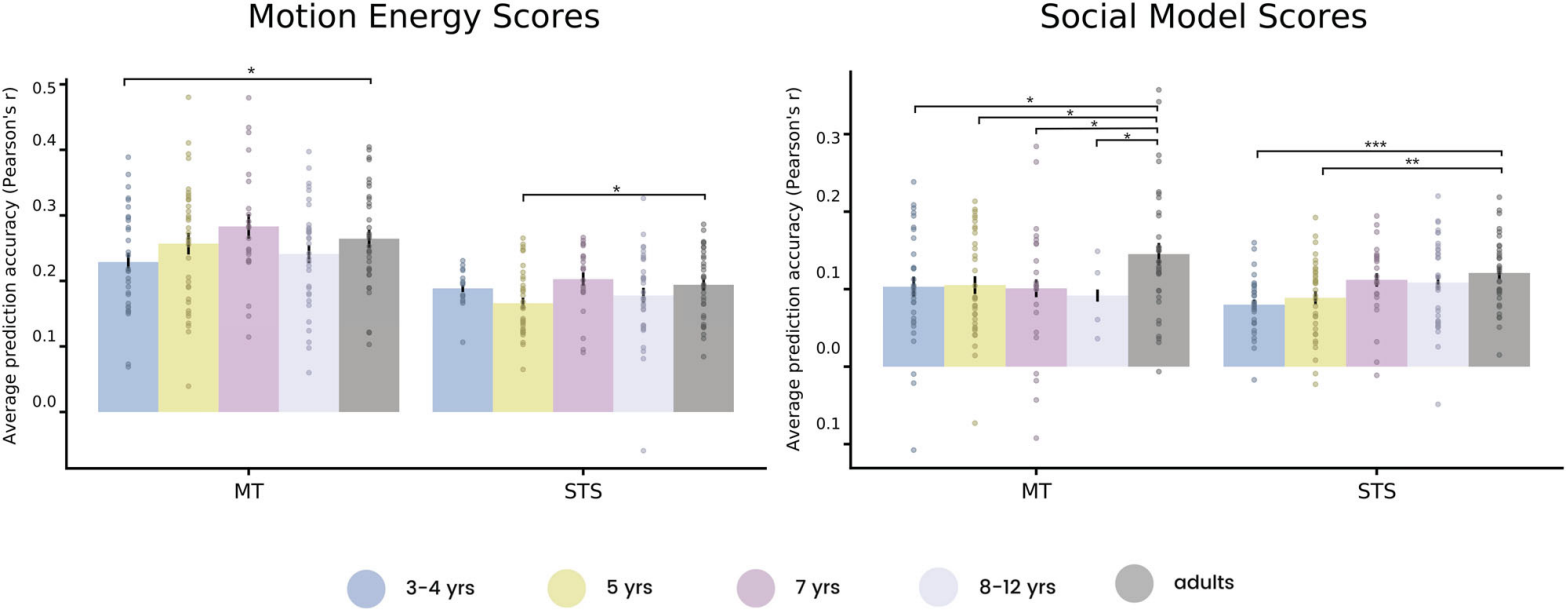
ROI encoding model results. Average voxel-wise prediction (Pearson's *r*) across subjects in each age group in MT and STS by motion energy features (left) and social features (right). Prediction is above chance (*p* < 0.05) in each ROI and age group for both models. Error bars represent standard error of the mean across subjects Asterisks indicate significantly different prediction between a child group and adults (*p < 0.05; **p < 0.01; ***p < 0.001) based on permutation testing (one-tailed, *n* = 5,000 permutations).

To investigate the development of motion and social feature representation across ages with more targeted tests that avoid the need for multiple-comparison correction, we turned to ROI analyses. In particular, we investigated responses in motion-sensitive region MT and an anatomical parcel encompassing both the TPJ and STS (referred to henceforth as STS). Both the visual and social-affective models could significantly predict activity in both ROIs in each age group. For the visual model, there were slight differences between adults and 3−4 and 5-year-old groups in MT and the STS, respectively (Fig. 4). Overall, however, visual responses looked largely similar to adults in both ROIs, suggesting that children have mature visual responses in these regions from an early age. The social-affective model predictions showed significant differences between adults and every group of children in MT and a significant difference between adults and the two youngest groups in STS, suggesting neural representations of social-affective features are developing throughout childhood. Notably, social-affective model scores were significantly above chance in all age groups.

### Early development of social interaction responses in the STS

Next, we sought to understand the representations of individual social features throughout development. We conducted our analysis in the combined TPJ and STS anatomical ROI. We looked at the predictivity of three key social features, faces, ToM, and social interaction, while controlling for all other features in the encoding model.

**Figure 5.**
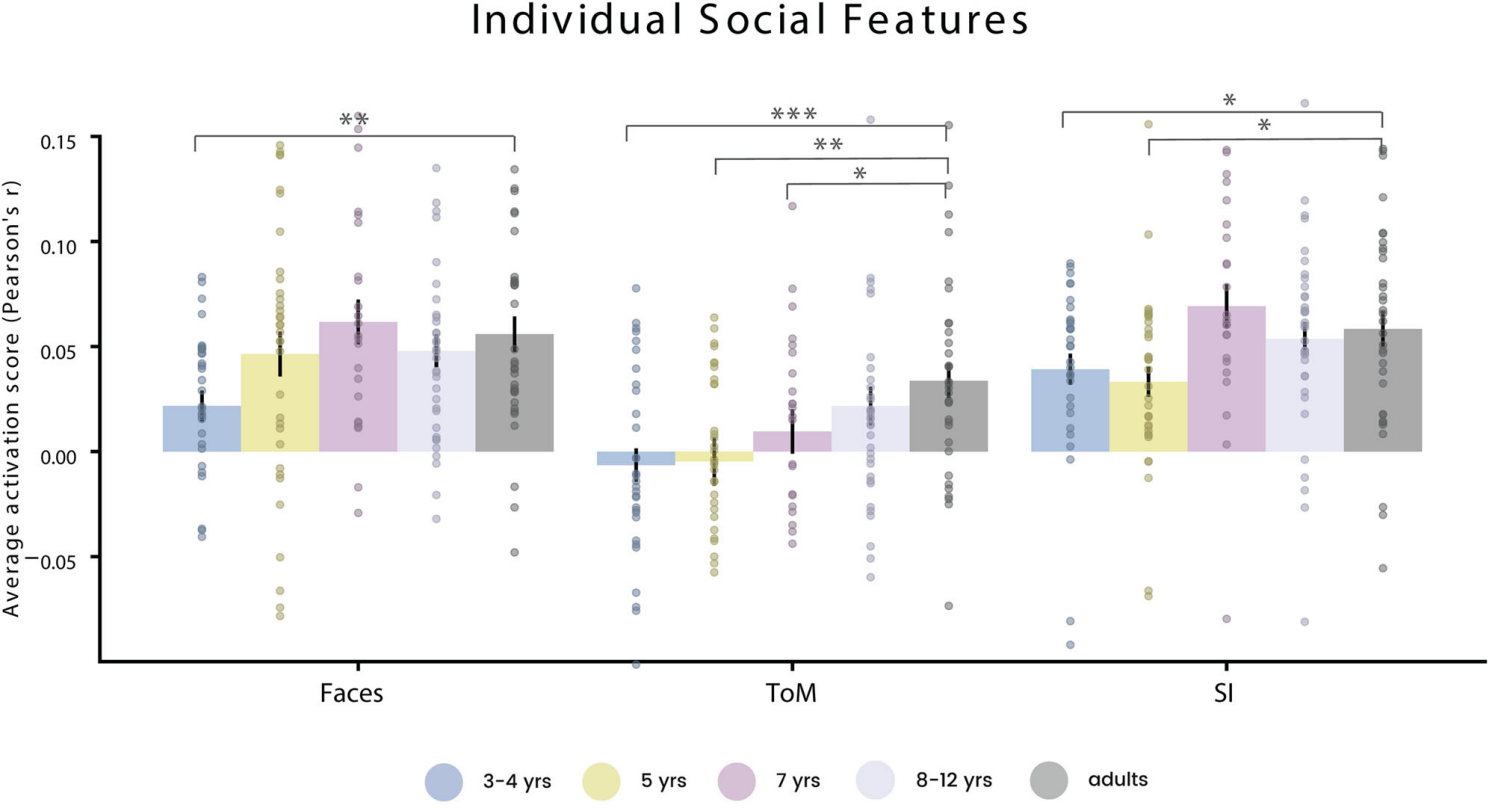
Individual social feature prediction accuracy in STS. Average prediction accuracy (Pearson's *r*) in STS across subjects in each age group by faces feature (left), ToM feature (middle), and social interaction features (right). Prediction is above chance (*p* < 0.05) in all age group for faces and social interaction model, but only in 8–12 years and adult groups for ToM. Error bars represent standard of error of the mean across subjects. Asterisks indicate significantly different prediction between a child group and adults (*p < 0.05; **p < 0.01; ***p < 0.001) based on permutation testing (one-tailed, *n* = 5,000 permutations).

Face features predicted significant responses in the STS in all age groups (Fig. 5, left). Though predictivity was similar across most age groups, there was a significant difference between adults and the 3–4-year-old group. ToM features significantly predicted responses in adults and the older children (Fig. 5, middle). Predictivity in 3–4 and 5 years was not above chance and significantly less than that of adults. Finally, social interaction features were significantly predictive of STS responses in all age groups (Fig. 5, right), including even 3–4-year-olds, with only minimal differences the youngest age groups and adults, suggesting that social interaction responses in the STS are present in children as young as 3.

## Discussion

Here we investigated the neural development of visual and social-affective feature representations in children 3–12 years old using a voxel-wise encoding approach. We found that an encoding model trained on only a handful of visual and social features could significantly predict whole-brain activity in even the youngest children. We also investigated predictivity based on individual social feature representations in the STS and found that social interaction features could significantly predict responses in all age groups, even when controlling for the effects of covarying visual and social features in the movie. Our results provide strong evidence that social interaction representations in the brain are early developing, similar to other perceptual abilities like motion and face processing (McMahon and Isik, 2023).

Interestingly, we find high predictivity of both motion features in the STS and social features in MT (Fig. 4). These results may be due to inherent confounds in the features of movie stimuli (Grall and Finn, 2022), though we aimed to control for these by quantifying the correlation between motion and social features (Fig. 2*b*) and fitting the regression with both feature spaces to “regress out” covarying effects of each model from the other model's prediction. Responses in STS are known to be highly sensitive to dynamic information (Pitcher et al., 2011), and it is possible that the multidimensional motion energy features capture variance in this region that cannot be accounted for by our simple binary social features. In MT, a growing body of work suggests that regions of the LOTC, including MT and surrounding regions, represent the sociality of actions (Wurm et al., 2017; Wurm and Caramazza, 2022). Our anatomical definition of MT may also include nearby regions in the extrastriate body area, and both of these regions have been identified as part of the recently proposed “lateral visual pathway” representing social motion (Pitcher and Ungerleider, 2021) and in particular social action features (McMahon et al., 2023). This work suggests that representations in LOTC and the lateral pathway are present early in childhood.

While prior studies have shown that biological motion selectivity in the STS is present in children and infants (Lloyd-Fox et al., 2011; Lisboa et al., 2020), these results provide the earliest evidence of social interaction-specific STS responses in children. One prior fNIRS study reported selectivity in mPFC but not STS when infants viewed videos depicting simple dyadic social interactions versus inverted interacting stimuli (Farris et al., 2022). The current study and prior studies with adults and older children (Isik et al., 2017; Walbrin et al., 2018, 2020) did not test inverted stimuli, so it is unknown whether the STS would show similar trends in adults or older children. However, we did not find strong predictivity for our social model in mPFC (Fig. 3). Importantly however, time course analyses in Farris et al. (2022) showed a strong selective response to social interactions over all other conditions in the first several seconds of their stimuli, with only later involvement of mPFC. It is possible the fast-changing nature of the video stimuli used here might explain this seeming discrepancy. Two prior studies have also identified social interaction-selective responses in the posterior STS in older children using point light stimuli (Sapey-Triomphe et al., 2017; Walbrin et al., 2020). Walbrin et al. found that while social interaction selectivity was present in children 6–12, selectivity continued to increase with age. In contrast, we did not see differences in social interaction predictivity between children in a similar age range (7 years and older) and adults, suggesting perhaps that the more engaging nature of our stimuli drive stronger results in children. However, the differences across the two studies in stimuli and analysis methods make it difficult to directly compare these results. Another important difference is that we did not have functional localizer data for our subjects and, therefore, used an anatomical ROI mask for the STS. Our STS mask was considerably larger than the functionally localized region used in the Walbrin study and may have limited our ability to see finer-grained developmental differences. The data used were also smoothed with a relatively large kernel size (5 mm) compared with some other voxel-wise encoding studies. Investigating developmental changes in movie responses in functionally localized regions and at a finer spatial scale with more data are interesting areas for future work.

Our results showed extremely similar motion predictivity between adults and all child groups, confirming prior reports of early developing motion responses (Biagi et al., 2023), and provide strong evidence that the other differences cannot simply be attributed to lower-quality data in children. We also find evidence for STS responses to faces in all age groups with slight differences between adults and 3–4-year-olds but adult-like predictivity in all other age groups. While some prior work has suggested that STS face selectivity does not develop until adolescence (Scherf et al., 2007), other studies using both fNIRS (Farroni et al., 2013) and fMRI (Kosakowski et al., 2023) have suggested that STS face selectivity is present in infants and adult-like in young children (Golarai et al., 2007). Our results lend further support to early development of face STS responses.

Our results are also consistent with the original study that collected this dataset showing that the functional specialization of the ToM network develops throughout childhood (Richardson et al., 2018), as well as prior studies showing reduced ToM selectivity in the TPJ for children compared with adults (Kobayashi et al., 2007; Saxe et al., 2009; Gweon et al., 2012). Unlike many of these studies, however, we do not find evidence for above-chance ToM responses in the TPJ/STS in children younger than 8 years old. There are several factors that could explain this discrepancy. First, the original Richardson study did not use event coding in their analysis, though they do conduct reverse correlation analysis and identify scenes evoking high mentalization during the peak responses in the ToM network. Second, our ToM feature had both the lowest inter-rater agreement and prevalence in the movie. Finally, and perhaps most likely, we used a “second-order” definition of ToM that defined events when a character on screen appeared to be thinking about another character's thoughts, goals, or emotions. We selected this criterion because it is easier to objectively label and has been shown to activate ToM regions in adults (Tholen et al., 2020; Lee Masson and Isik, 2021), but it is possible that young children do not pick up on these more subtle social cues.

In sum, we showed for the first time that a voxel-wise encoding model approach can explain fMRI responses and isolate the specific contributions of different perceptual and social features in young children using only a few minutes of data. Our findings for the first time showed evidence of STS social interaction responses in children as young as 3. Our results also confirmed prior reports of early developing face and motion responses and later developing ToM responses. We believe these results as well as the methodological framework can open doors to future content-based fMRI movie studies of many cognitive functions in young children and other difficult-to-scan populations.

### Code accessibility

The current study used analysis code from Nunez-Elizalde AO, Huth AG, and Gallant JL, 2019 (https://github.com/gallantlab/tikreg) as well as custom python scripts available on GitHub (https://github.com/Isik-lab/partly_cloudy_encoding).
